# Pediatric Septoplasty: Benefits, Challenges, and Clinical Recommendations—Comprehensive Review of Young ESPO

**DOI:** 10.3390/jcm14155537

**Published:** 2025-08-06

**Authors:** Jakub Zieliński, Sara Costa, Maryana Cherkes, Natalia Glibbery, Petra Kovács, Luiza Mitrea-Sirețeanu, Marek Ciller, Miray-Su Yılmaz Topçuoğlu

**Affiliations:** 1 Department of Otolaryngology, Head and Neck Surgery, Wroclaw Medical University, 50-367 Wroclaw, Poland; 2 Department of Otolaryngology-Head and Neck Surgery, Unidade Local de Saúde de Santo António, 4050-342 Porto, Portugal; saraprvcosta@gmail.com; 3 Department of Otorhinolaryngology & Head and Neck Surgery, Medical University of Graz, Auenbruggerplatz 26, 8036 Graz, Austria; maryana.cherkes@gmail.com; 4 Department of Otolaryngology, West Suffolk Hospital, Bury St Edmunds IP33 2QZ, Suffolk, UK; nat.glibbery@gmail.com; 5 Department of Otolaryngology, Heim Pál National Pediatric Institute, 1089 Budapest, Hungary; dr.med.kovacs.petra@gmail.com; 6 Saint Mary Plaza Clinic, Bulevardul Timisoara, Nr. 26 Z, Sector 6, 061331 Bucharest, Romania; luiza.sireteanu@gmail.com; 7 Department of Otorhinolaryngology, Second Faculty of Medicine, University Hospital Motol, V Úvalu 84, 150 06 Prague, Czech Republic; marek.ciller@gmail.com; 8 Department of Otorhinolaryngology, Head and Neck Surgery, University Hospital Heidelberg, Im Neuenheimer Feld 400, 69120 Heidelberg, Germany; miray-su.yilmaztopcuoglu@med.uni-heidelberg.de

**Keywords:** pediatric, septoplasty, congenital septal anomalies, pediatric surgery

## Abstract

This comprehensive review examines the role of septoplasty in the pediatric population, emphasizing its therapeutic significance in relieving nasal obstruction and facilitating normal craniofacial growth. Despite the evident advantages of septoplasty, its application in young patients remains a subject of ongoing debate. This issue is primarily characterized by concerns regarding the still-developing immaturity of nasal cartilage, potential intraoperative and postoperative risks, and the current absence of robust data on long-term outcomes following septoplasty. Common complications such as bleeding, septal perforation, saddle nose deformity, and persistent nasal obstruction are reported in the literature; however, many studies lack long-term follow-up data on the incidence of these adverse events and revision rates, which may be higher compared to adult populations, often leading to the need for secondary surgical interventions. Strict inclusion criteria and comprehensive patient selection are paramount to maximize therapeutic success while minimizing complications. Current evidence suggests that appropriately indicated septoplasty can improve airway patency, support optimal facial development, and reduce the risk of secondary sinonasal pathology. There is a significant necessity for additional prospective, large-scale studies to establish standardized therapeutic guidelines and management strategies for this specific population, thereby ensuring effective and evidence-based pediatric otolaryngologic care.

## 1. Introduction

Nasal septal surgery, commonly termed septoplasty, is a procedure aimed at correcting deviations of the nasal septum to improve nasal airflow and function and to address associated aesthetic concerns [1]. The history of septal surgery dates back centuries, exemplified by early descriptions in ancient Indian and Egyptian medical texts and records. The Ebers Papyrus, dating back to approximately 3500 BC in Egypt, is historically recognized as one of the earliest references to nasal and rhinologic procedures. The 18th century brought some recognition of the importance of manual techniques for correcting septal deformities. In 1757, Quelmatz recommended daily digital pressure on the septum as a conservative method to manage deformities, reflecting an understanding of the flexibility and potential for manual correction even before surgical intervention was formalized [2]. Later, in 1875, Adams proposed mechanically fracturing and splinting the nasal cartilage to correct deviations, emphasizing the mechanical approach to septal correction [3]. The late 19th century marked a significant evolution in septoplasty surgery with the introduction of more definitive surgical techniques. The Bosworth operation, common in the United States during this period, involved resecting deviated sections of the septum, aiming to correct nasal obstruction [4]. However, this method often yielded suboptimal results. In 1882, Ingals pioneered the modern concept of septal surgery by introducing en bloc resection of small parts of the septal cartilage, which set the groundwork for contemporary septoplasty techniques [5]. Around the same time, the introduction of cocaine as a local anesthetic significantly improved surgical conditions, allowing longer and more refined operations with better hemostasis [6]. At the turn of the 20th century, Freer and Killian developed and refined the submucous resection (SMR), a procedure that involves elevating mucoperichondrial and mucoperiosteal flaps to resect or straighten the deviated cartilage and bone of the septum [7,8]. These surgeries became the basis of modern septoplasty, emphasizing minimal trauma and preserving nasal support structures. The correction of deviations involving the caudal septum proved more challenging. In 1929, Metzenbaum explored techniques to manipulate and reposition the caudal septum, advocating for the use of the swinging door technique [9]. By 1937, Peer advocated for removing and straightening the caudal septum, then repositioning it centrally [10]. In 1947, Cottle introduced a conservative approach emphasized by the hemitransfixion incision [11]. His work highlighted the importance of limiting resection to avoid deformities such as dorsal saddling, retraction of the columella, and alar widening, which had been observed in patients undergoing more extensive resections. Cottle promoted preserving as much nasal support as possible, marking a significant step toward modern, conservative septal correction philosophies [11]. These historical developments reflect a progressive shift from aggressive, resective procedures towards more refined, tissue-preserving techniques emphasizing function, aesthetics, and stability, many of which form the basis of current septal surgery practices.

A deviated nasal septum refers to an abnormal curvature or displacement of the cartilaginous and/or bony components of the nasal septum, leading to partial or complete obstruction of nasal airflow. The deviation can be congenital or acquired. Congenital deviations often occur during fetal development, typically attributable to genetic predisposition and in utero factors influencing facial growth, such as amniotic bands or intrauterine trauma. Acquired deviations are frequently due to nasal or facial trauma, which is a common cause in children [12,13]. Traumatic causes are prevalent in children due to falls, sports injuries, or accidental impacts, particularly during early developmental stages when the nasal bones and cartilage are more susceptible to injury [14]. Nasal septal surgery before or during adolescence has been a subject of controversy due to concerns about performing procedures on a developing nose. The primary concern involves the potential disruption of midfacial growth and the risk of long-term functional and aesthetic sequelae. As a result, the prevailing approach for much of the past century has been to defer surgical intervention until skeletal maturity, except in cases of significant functional impairment or deformity [15]. In recent years, however, this long-standing paradigm has been increasingly challenged. Advances in the understanding of nasal and midfacial growth have led to new insights, suggesting that nasal surgery may be safely performed in pediatric patients when conservative techniques are employed. These approaches preserve key anatomical structures, such as the sphenodorsal and sphenospinal cartilage zones, which function as growth centers guiding craniofacial development [16]. A recent systematic review concluded that septoplasty in pediatric patients does not appear to have a detrimental effect on midfacial growth based on current evidence [17]. This evolving perspective is further supported by evidence linking nasal obstruction itself to a range of developmental consequences. Nasal obstruction in children has been associated with sleep-disordered breathing, persistent nasal congestion, and impaired quality of life, including reduced attention span, cognitive dysfunction, and disrupted sleep patterns [18]. Additionally, chronic nasal obstruction often leads to obligate mouth breathing, which has been implicated in the development of craniofacial abnormalities and dental malocclusion [19]. A 2021 systematic review and meta-analysis further demonstrated that chronic mouth breathing in children is associated with craniofacial changes, including jaw underdevelopment, backward and downward rotation of the maxilla and mandible, steep occlusal planes, and labial inclination of the upper anterior teeth [20]. Despite this growing body of evidence, controversy remains regarding the optimal timing and indications for nasal septal surgery in pediatric patients. At present, there is no clear consensus on the severity of nasal obstruction that warrants surgical intervention. This review focuses on original research articles published from 2014 onward to capture the most recent advances in surgical techniques, diagnostic methods, and outcome assessments. This approach ensures exclusion of outdated studies that relied on subjective questionnaires, older imaging modalities, and diagnostic criteria that have since been superseded. By restricting the search to articles published in the last decade, we aim to provide an up-to-date synthesis capturing contemporary surgical practices, understanding of growth-related risks, and outcome measures. Given that pediatric septoplasty is a relatively niche subject with scarce dedicated research, the available literature comprises primarily small case studies, retrospective analyses, and limited prospective studies. The objective of this review is to evaluate the current evidence regarding the efficacy and safety of septal surgery in children and adolescents with nasal obstruction.

## 2. Background and Methods

Pediatric septoplasty, which involves surgical correction of deviated nasal septum in children and adolescents, remains a relatively under-investigated area within rhinology and pediatric otolaryngology. Despite the widespread recognition of the importance of the nasal septum in airway patency, there is a paucity of contemporary, high-quality evidence regarding the indications, safety, and long-term outcomes of septoplasty in this age group. Septoplasty in pediatric patients remains challenging due to variability in presentation, diagnostic tools, and an evolving understanding of the impact of surgical intervention on facial growth and development. The search was limited to articles published from January 2014 to the present to ensure inclusion of the most recent and relevant studies. The literature review was conducted using the PubMed database, using a combination of keywords “pediatric septoplasty,” “congenital septal anomalies,” “septal deviation in children,” “midfacial growth,” and “craniofacial development.” These keywords were chosen to encompass the core topics of interest, emphasizing not only surgical intervention but also long-term craniofacial development outcomes.

In addition to the main keywords, Boolean operators such as AND, OR, and NOT were employed to refine the search. For example, combinations like “pediatric septoplasty” AND “congenital septal anomalies” were used to narrow down results to studies that explicitly addressed surgical intervention in children. The initial search resulted in a total of 48 articles, which included original research articles, case series, and observational studies. The inclusion criteria for selection were narrowly defined to ensure the relevance and quality of the evidence. Articles were included if they met the following criteria: the study population included patients under 18 years of age undergoing septoplasty or septorhinoplasty for septal deviation; the study provided objective clinical outcomes related to the surgical procedure, with assessments of airway patency, nasal function, or cosmetic appearance; the article was published in English. Our review included only original studies, resulting in 18 that met the inclusion criteria.

## 3. Results

The results of this review are based on a comprehensive analysis of included studies. For clarity and coherence, the findings are organized into distinct subsections. This structured approach underscores existing knowledge gaps, highlights the need for standardized reporting, and provides a framework to guide future research aimed at improving the understanding and management of pediatric septoplasty outcomes.

### 3.1. Population Size, Age, and Sex Distribution

A total of 18 studies were included in the review [21,22,23,24,25,26,27,28,29,30,31,32,33,34,35,36,37,38], comprising a combined sample of 28,638 pediatric patients. Of these, 12 (63.15%) reported sex distribution data, encompassing 25,946 patients—15,696 patients (60.49%) were boys and 10,250 (39.51%) were girls [21,22,23,24,25,26,30,31,35,36,37,38].

Information on age distribution—reported as mean age, median age, or age range—was available for the majority of studies. The extracted data were systematically organized into Table 1, which summarizes the relevant details. The youngest and oldest patients reported across the included cohorts were 3 and 18 years of age, respectively. Although individual patient-level data were unavailable, the reported ranges and averages suggest that most studies focused primarily on school-aged children and early adolescents, with the most commonly reported age interval ranging from approximately 6 to 16 years. Due to heterogeneity in reporting and the reliance on summary measures rather than raw data, precise reconstruction of the overall age distribution was not feasible.

### 3.2. Preoperative Symptoms and Diagnostics

**Figure 1:**
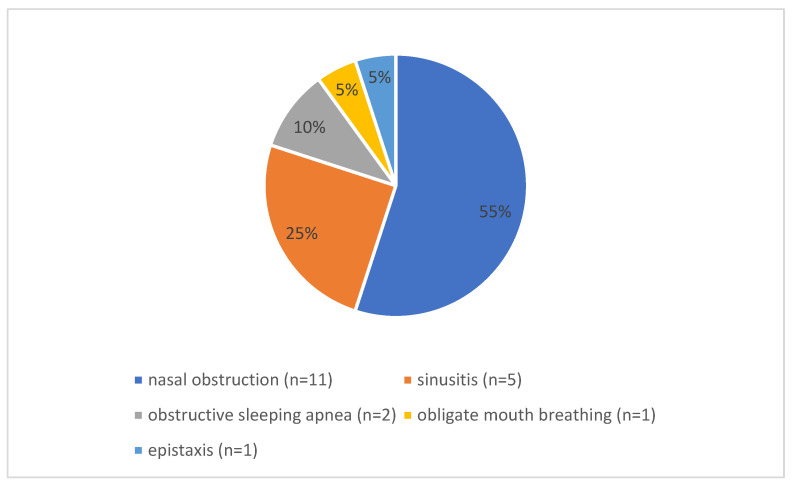
Distribution of preoperative symptoms. The pie chart shows the distribution of preoperative symptoms that led to the indication for pediatric septoplasty in the papers that were included in this study and discussed preoperative symptoms (n = 13).

Among the included articles, 14 out of 18 reported the predominant preoperative symptoms in pediatric patients undergoing septoplasty (Figure 1). The most commonly cited symptoms were nasal obstruction (n = 11) [21,22,23,24,26,28,29,30,31,32,33], recurrent sinusitis (n = 5) [21,24,26,29,32], obstructive sleep apnea (n = 2) [26,32], obligatory mouth breathing (n = 1) [28], and epistaxis (n = 1) [26]. In the analyzed literature, nasal obstruction was measured using preoperative nasal examination findings, such as internal and external nasal valve narrowing, using peak nasal inspiratory flow, rhinomanometry, and preoperative CT imaging of the nose and paranasal sinuses. However, the articles do not provide any description of a specific diagnosis of obstructive sleep apnea. Similarly, 14 studies described the indications for surgical intervention. The primary indications for surgical intervention included diminished quality of life [21,22] due to symptomatic septal deviation causing nasal obstruction [21,22,30,31,32,38] or recurrent sinusitis [21,31], both refractory to conservative management [24,32]. Clinical examination frequently revealed significant congenital or traumatic septal deviations [23,24,28,29,30,32,33,34,38], often accompanied by high Nasal Obstruction Symptom Evaluation (NOSE) [21,33]. The NOSE score is a subjective measure based on patient self-assessment rather than an objective criterion.

**Figure 2:**
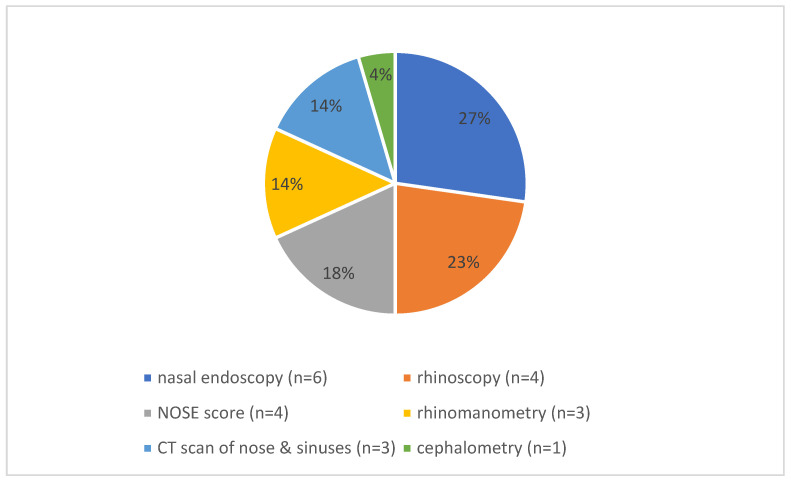
Distribution of preoperative diagnostics. The pie chart shows the distribution of preoperative symptoms that led to the indication for pediatric septoplasty in the papers that were included in this study and discussed preoperative symptoms (n = 13).

Eight of the eighteen studies addressed preoperative diagnostic assessments (Figure 2). Common preoperative diagnostic modalities included nasal endoscopy (n = 6) [21,24,31,32,33,38], rhinoscopy (n = 5) [30,31,32,33,38], the NOSE score (n = 4) [28,32,33,38], rhinomanometry (n = 3) [28,32,33], preoperative CT imaging of the nose and paranasal sinuses (n = 3) [24,31,33], and cephalometry (n = 1) [28].

### 3.3. Septoplasty Techniques, Adjunct Procedures, and Postoperative Care

Across the original studies analyzing septoplasty procedures, a variety of surgical techniques and adjunctive interventions were reported, reflecting both established and evolving practices in pediatric nasal septal surgery. Surgical approaches included both closed (endonasal) and open septoplasty techniques [24,33]. Open septoplasty was described as unilateral mucoperichondrial flap elevation and excision of a cartilage strip. Unilateral mucoperichondrial flap elevation refers to the surgical technique of raising a flap of tissue (mucoperichondrium) from one side of the nasal septum, leaving the other side undisturbed. It allows access to the cartilage and bone of the septum for reshaping and straightening, while preserving the lining on the opposite side.

Functional septorhinoplasty was described in multiple studies [22,31,32], frequently involving osteotomies, spreader graft placement, dorsal hump reduction, and columellar strut insertion. However, only the study of Fuller et al. [32] primarily utilized an open approach for functional septorhinoplasty in adolescent patients, with the majority of girls likely having completed their growth spurt. The researchers acknowledged the importance of performing surgery with conservative techniques that avoid disruption of key structures. Ghosh et al. [22] emphasized the importance of preserving anatomical landmarks, including the mucoperichondrium and growth centers, with reimplantation of straightened septal cartilage. Manteghi et al. [31] employed cartilage-sparing techniques when feasible and reported adjunctive procedures such as turbinate reduction (35.8%) and concha bullosa excision (13.5%). Lee et al. utilized both open and closed approaches, with cartilage preservation strategies and the use of quilting sutures or silastic splints depending on the surgical technique [34].

Postoperative splinting and packing practices varied considerably across studies. Most authors reported the use of internal nasal splints, typically secured with absorbable sutures and removed between 2 and 14 days postoperatively [21,33]. When nasal packing was employed, materials included Merocel [28,30] and ribbon gauze. Ori et al. [28] utilized bilateral Merocel packs, which were removed on postoperative day one, consistent with their minimally invasive “quick” septoplasty technique. In contrast, Lee et al. [34] avoided nasal packing entirely in the closed approach, instead employing quilting sutures for stabilization.

Turbinoplasty and inferior turbinate reduction were performed with variable frequency across studies. The highest reported rate was 92%, where partial inferior turbinectomy was conducted when indicated [36]. Benyo et al. [23], Fuller et al. [32] (12.8%), and Maniglia et al. [37] (33.7%) also reported concurrent turbinate procedures. Inferior turbinate cauterization was performed in 92% of cases in one study [25]. In contrast, several authors explicitly excluded patients requiring turbinate surgery, reflecting variations in study protocols [28,35].

Overall, the reviewed studies demonstrated a spectrum of surgical approaches, ranging from conservative septoplasty techniques that emphasize growth center preservation in pediatric patients [30,36] to more complex reconstructive strategies employed in functional septorhinoplasty contexts [31,32]. Across this spectrum, consistent principles included mucosal preservation, cartilage-sparing techniques, and individualized adjunctive procedures.

### 3.4. Complications

**Figure 3:**
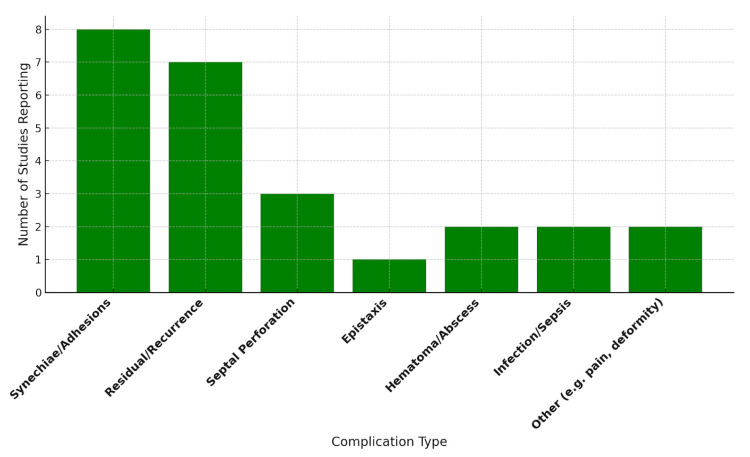
Reported complication types across the reviewed studies.

Postoperative complications varied in frequency and type across studies (Figure 3). The most commonly reported complications included synechiae/intranasal adhesions, described in eight studies [22,25,28,30,33,36,37,38]; residual or recurrent septal deviation, reported in seven studies [21,28,30,33,36,37,38]; septal perforation, noted in three studies [21,29,30]; and epistaxis, highlighted in one study [29]. Septal hematoma or abscess was identified in two studies [32,36], while systemic infection or sepsis was reported in large cohort analyses [23,27]. The overall incidence of complications was generally low, with most cases being self-limiting or managed conservatively.

**Figure 4:**
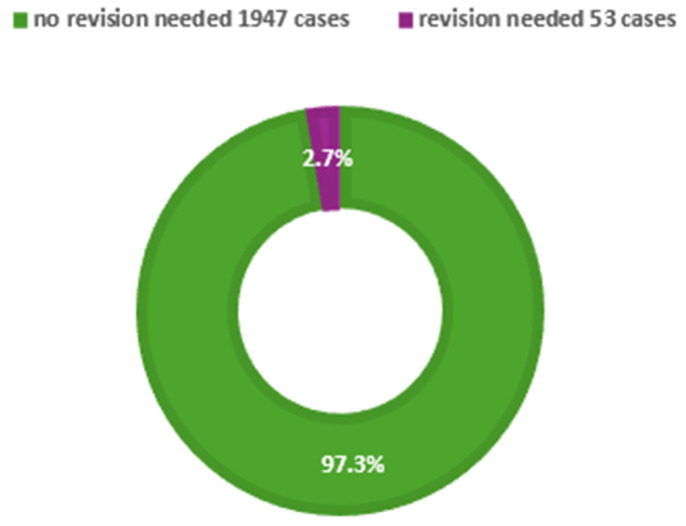
Revision rates across the reviewed studies.

Seven of the eighteen studies reported revision surgery rates (Figure 4). Reported revision rates ranged from 0% to 14%. Shah et al. [26] documented 704 revision surgeries, corresponding to a 2.9% revision rate within a cohort of 24,322 children, although specific details regarding complications were not provided. The large cohort includes only the data related to the collected information and does not specify the reasons for revision or provide objective functional parameters. Bishop et al. [29] observed higher revision rates among younger children, with a rate of 14% in patients under 14 years of age. Other reported revision rates included 4.45% [37], 5.1% [32], 0.4% [23], and 4% [30]. Yilmaz et al. [38] reported no revision cases. Several studies [22,24,28,31] did not provide information on revision outcomes. Notably, many studies lacked detailed reporting on long-term outcomes such as facial growth disturbances or nasal developmental issues.

### 3.5. Craniofacial Growth and Development

Among all the studies reviewed, only four explicitly assessed the impact of septoplasty on facial growth and development. In one prospective study [28], septoplasty was performed on 111 children aged 6 to 13 years, with cephalometric measurements obtained preoperatively and again at 18 years of age. Objective measurements demonstrated favorable changes in gonial angle and significant increases in the ratio of upper to total anterior facial height with subsequent spontaneous correction of the partial midface hypoplasia found preoperatively. Additional findings included increased maxillary intermolar width and a reduced prevalence of crossbite, with no evidence of impaired facial growth.

Abdelaal et al. [24] evaluated 39 patients who underwent endoscopic septoplasty prior to 17 years of age. Lateral cephalometric analysis performed at age 17 revealed no significant differences between these patients and age-matched controls in palatal length, anterior skull base length, midface protrusion, or midface length.

Similarly, two further studies reported no aesthetic abnormalities or facial growth disturbances during follow-up periods of 6 to 12 months; however, these assessments were based on clinical observation without objective measurements [21,36].

### 3.6. Outcomes

Of the 18 studies included in this review, 14 evaluated pediatric septoplasty outcomes using either objective or subjective assessment methods. Only three studies employed objective measures: combined anthropometric assessments (cephalometric and orthodontic measurements) and anterior active rhinomanometry [28]; peak nasal inspiratory flow, which increased from 66.2 L/min preoperatively to 90.8 L/min postoperatively [32]; and rhinomanometry, which demonstrated a postoperative reduction in nasal resistance, and a positive correlation was identified between preoperative nasal resistance (reflecting obstruction severity) and postoperative quality-of-life improvement [33]. The majority of studies relied on subjective measures, most commonly the NOSE scale, visual analog scale (VAS), or other quality-of-life questionnaires, such as SN-5, HRQoL, and EQ5D. For example, Ghosh et al. [22] reported a significant reduction in NOSE scores (from 72 to 22; *p* < 0.05), while Fuller et al. [32] observed an improvement in EQ5D VAS scores (from 76.2 to 85.8; mean change 9.6; *p* = 0.056).

Overall, the outcomes of pediatric septoplasty appear favorable, as demonstrated by both objective and subjective measures. None of the included studies reported any negative impact on facial growth, supporting the safety and efficacy of the procedure from both developmental and functional perspectives.

## 4. Discussion

This research thoroughly reviews pediatric septoplasty, covering safety, techniques, growth concerns, and outcomes. Overall, despite some variation across studies, the evidence generally supports septoplasty as a safe option for children with nasal obstruction due to septal deviation.

The included studies comprise a large cohort of 28,638 pediatric patients, predominantly male at 60.49%, with ages ranging from 3 to 18 years. Most studies focused on school-aged children and early adolescents, particularly those between 6 and 16 years of age. Although the absence of detailed individual-level age data restricts precise subgroup analysis, understanding the distribution of age and sex is essential for risk assessment and surgical technique. The skewed sex ratio highlights the need for further research into sex-specific anatomical or developmental factors influencing the prevalence of septal deviation and surgical outcomes.

The predominant presenting symptom was nasal congestion, consistent with broader epidemiological data on nasal disorders in the pediatric population. Other significant symptoms included recurrent sinusitis, sleep-disordered breathing, mouth breathing, and epistaxis. Surgical intervention is generally indicated for patients with substantial symptomatic impairment unresponsive to conservative management, emphasizing the importance of symptom severity and its impact on quality of life [21,22]. Notably, while nasal obstruction was primarily assessed through clinical examination—such as internal and external nasal valve inspection—and objective measures like peak nasal inspiratory flow, specific diagnostic criteria for obstructive sleep apnea were rarely detailed across studies. This inconsistency underscores a gap in the standardized evaluation of sleep-disordered breathing in this population.

Preoperative diagnostic evaluations varied across studies but commonly included endoscopy, rhinoscopy, and imaging modalities such as CT scans. In some studies, additional assessments such as rhinomanometry and the NOSE score were employed to enhance both objective and subjective evaluation of nasal patency [28,33]. This multimodal approach underscores the importance of comprehensive preoperative assessment to guide surgical planning and monitor postoperative outcomes.

A broad range of surgical approaches was reported, reflecting both conventional and evolving techniques. Closed (endonasal) and open septoplasty approaches were utilized, with endoscopic techniques increasingly employed due to their advantages in tissue preservation and enhanced visualization. In cases involving concomitant deformities, functional septorhinoplasty—incorporating osteotomies, grafting, and turbinate procedures—was frequently performed [22,31,32]. The reviewed studies underscore considerable variability in surgical techniques and adjunctive interventions employed in pediatric septoplasty, reflecting both the evolution of minimally invasive approaches and ongoing debates regarding optimal management strategies. While approaches ranged from traditional open and closed septoplasty procedures to more conservative cartilage-sparing techniques, a critical appraisal reveals that many studies lack standardized protocols, which complicates the comparison of outcomes and the formulation of evidence-based guidelines.

In accordance with pediatric surgical principles that prioritize growth center preservation, mucosal preservation and cartilage-sparing techniques were emphasized to minimize growth disturbances [30,36]. Adjunctive procedures such as concha bullosa excision and turbinate reduction were frequently performed and tailored to individual anatomical variations, demonstrating improvements in nasal airflow outcomes [23,31]. The heterogeneity in inclusion criteria—particularly regarding turbinate procedures—reflects a broader issue: many studies lack clarity on how surgical candidacy is determined. Turbinate reduction (turbinoplasty) is frequently performed as an adjunct to septoplasty to improve nasal airflow and address nasal obstruction comprehensively. The analyzed studies encompass both procedures, as they are often combined in clinical practice. However, turbinoplasty is a distinct procedure from septoplasty. The authors acknowledge that it should be studied separately to avoid conflating the two interventions and to better understand their individual effects and indications.

Postoperative management techniques varied widely across studies, including the use of internal splints and nasal packing materials. Recent guidelines favor early splint removal (within 2 to 14 days) and the use of absorbable splints to reduce patient discomfort and minimize complications [28,33]. When feasible, the avoidance of nasal packing further contributes to improved patient comfort.

A major concern in pediatric septoplasty involves its potential impact on craniofacial development. With limited data—including prospective studies and assessments focusing on facial and nasal measurements, as well as functional evaluations such as rhinometry and rhinomanometry—Ori et al. and Abdelaal et al. [24,28] show no clear evidence of growth restriction following conservative septoplasty. In addition to improvements in crossbite frequency, longitudinal assessments demonstrated postoperative normalization of cephalometric parameters, including the gonial angle and facial height ratios, suggesting potential benefits of early surgical intervention. Cephalometry, a radiographic imaging technique that provides detailed measurements of craniofacial structures, can serve as an objective tool in evaluating nasal function and airflow. By analyzing specific anatomical parameters—such as the size and shape of the nasal cavity, nasal septum deviation, and the dimensions of the choanae—cephalometry can help identify structural obstructions or deformities that may impair nasal airflow. Although it does not directly measure airflow, cephalometric measurements can be correlated with functional assessments, aiding in the diagnosis of nasal obstruction. For example, a narrowed nasal valve area or septal deviation identified through cephalometry can suggest compromised nasal patency, guiding clinical decision-making and treatment planning. When combined with other objective tests like rhinomanometry or acoustic rhinometry, cephalometry provides a comprehensive evaluation of nasal anatomy and function.

Abdelaal et al. [24] also found no significant differences in midfacial development at age 17 between patients who underwent operation and control subjects, further supporting the safety of septoplasty in growing children when performed appropriately. These findings support the hypothesis that alleviating nasal obstruction—and thereby reducing obligatory mouth breathing—may positively influence dentofacial development, addressing previous concerns regarding surgical interference with growth centers [17].

Pediatric septoplasty is generally associated with a low complication rate, with most adverse events being minor and manageable. The most commonly reported complications included intranasal synechiae, residual deviation, and recurrent symptoms, which occasionally required revision surgery at rates ranging from 0.4% to 14% [30,37]. Notably, higher revision rates were observed in children under 14 years of age, potentially attributable to technical limitations, ongoing craniofacial growth, or elevated expectations for surgical outcomes. Major complications—such as septal hematomas, systemic infections, or septal perforation—were rare. Large cohort studies further confirmed the safety profile of the procedure, reporting low 30-day readmission and reoperation rates [23,27]. However, detailed information regarding the frequency of complications, revision rates, and long-term outcomes was often incomplete or entirely absent in the reviewed studies. On the other hand, the study performed by Dąbrowska-Bień et al. [39] offers a valuable insight into the complications of septoplasty. Among 5639 patients, complications occurred in 3.42%, with excessive bleeding being the most frequent (3.3%). Septal perforation was observed in 2.3% of cases. Hyposmia (reduced sense of smell) and infection or prolonged healing were each reported in 3.1% of patients, generally resolving within months. Notably, the article provides a detailed analysis of complications, a rarity in septoplasty research. Future studies should emulate this comprehensive approach, as standardized complication reporting is essential for improving patient outcomes and surgical techniques.

## 5. Conclusions

Recent evidence indicates that pediatric septoplasty, when performed using minimally invasive cartilage-preserving techniques, can safely alleviate significant nasal obstruction without compromising craniofacial growth. However, the current literature predominantly comprises small, retrospective studies with inconsistent reporting of long-term outcomes, underscoring the need for standardized, prospective research. Clinically, surgeons should prioritize conservative approaches that maintain growth centers and consider adjunct procedures like turbinate reduction to optimize airflow. Importantly, careful patient selection—focusing on children with severe, refractory symptoms—is crucial to balance benefits against potential risks. Future investigations should aim to develop evidence-based guidelines, which are essential in septoplasty for children to ensure safe, effective, and standardized care. They help clinicians make informed decisions based on the best available scientific evidence, minimize variability in treatment approaches, and improve patient outcomes. Implementing guidelines to pediatric patients is particularly important due to their unique anatomical and developmental considerations. This can optimize surgical results while reducing the risk of complications. This targeted approach will help refine indications and maximize safety and efficacy, thereby advancing pediatric septoplasty from an empirical procedure to a standardized, evidence-supported intervention.

## Figures and Tables

**Table 1:** Population size, age, and sex distribution.

Authors	Population (n)	Male (n)	Female (n)	Range of Age (Years)	Mean Age
Alshehri et al. [21]	29	13	16	-	9.45
Ghosh et al. [22]	25	14	11	8–14	11.5
Benyo et al. [23]	729	503	226	6–16	-
Abdelaal et al. [24]	39	30	9	12–15.8	14.2
Yaseen et al. [25]	100	60	40	3–18	-
Shah et al. [26]	24,322	14,618	9704	≤18	-
Raghavan et al. [27]	2290	-	-	-	14.2
Ori et al. [28]	111	-	-	6–13	9.4
Bishop et al. [29]	194	-	-	-	14.6
Koirala [30]	50	37	13	-	12.78
Manteghi et al. [31]	136	94	42	-	15.7
Fuller et al. [32]	39	-	-	7–18	15.9
Sabry et al. [33]	30	-	-	5–17	12.7
Lee et al. [34]	28	-	-	-	-
Anderson et al. [35]	29	16	13	5–16	11.2
Matin et al. [36]	250	163	87	7–14	-
Maniglia et al. [37]	202	124	78	4–16	-
Yilmaz et al. [38]	35	24	11	8–16	13.4
**Total population**	28,638				
**Sex distribution**	25,946	15,696	10,250		

## Data Availability

No new clinical data were created. Data are available in a publicly accessible repository at https://pubmed.ncbi.nlm.nih.gov/, accessed on 1 June 2025.

## Abbreviations

The following abbreviations are used in this manuscript:

ESPO	European Society of Pediatric Otorhinolaryngology
NOSE	Nasal Obstruction Symptom Evaluation
